# Up regulation of Rho-associated coiled-coil containing kinase1 (ROCK1) is associated with genetic instability and poor prognosis in prostate cancer

**DOI:** 10.18632/aging.102294

**Published:** 2019-09-25

**Authors:** Stefan Steurer, Benjamin Hager, Franziska Büscheck, Doris Höflmayer, Maria Christina Tsourlakis, Sarah Minner, Till S. Clauditz, Claudia Hube-Magg, Andreas M. Luebke, Ronald Simon, Jakob R. Izbicki, Eike Burandt, Guido Sauter, Christoph Fraune, Sören Weidemann, Thorsten Schlomm, Hans Heinzer, Alexander Haese, Markus Graefen, Hartwig Huland, Asmus Heumann

**Affiliations:** 1 Institute of Pathology, University Medical Center Hamburg-Eppendorf, Hamburg, Germany; 2 General, Visceral and Thoracic Surgery Department and Clinic, University Medical Center Hamburg-Eppendorf, Hamburg, Germany; 3 Martini-Clinic, Prostate Cancer Center, University Medical Center Hamburg-Eppendorf, Hamburg, Germany; 4 Department of Urology, Charité - Universitätsmedizin Berlin, Berlin, Germany

**Keywords:** ROCK1, prostate cancer, prognosis, immunohistochemistry, tissue micro array

## Abstract

Background and objectives: Overexpression of the cytoskeleton-modulating kinase ROCK1 has been associated with unfavorable outcome in many cancers, but its impact in prostate cancer is largely unknown.

Results: A weak ROCK1 staining was found in >90% of normal, and cancerous prostate tissues, but was generally stronger in cancer cells as compared to adjacent normal glands. In cancer, ROCK1 staining was considered weak, moderate, and strong in 22%, 53%, and 18% of cases respectively. Higher ROCK1 expression levels were associated with tumor stage, and Gleason grade, positive nodal stage, positive surgical margin, accelerated cell proliferation and early PSA recurrence in multivariable analysis. ROCK1 up regulation was associated with androgen receptor (AR) expression, *TMPRSS2:ER*G fusion, genomic deletions of the PTEN tumor suppressor, as well as recurrent deletions at chromosomes 3p, 5q, 6q. Strong ROCK1 staining was found in 3% of AR-negative, but in 27% of strongly AR positive cancers, in 13% of ERG-negative but in 25% of ERG positive cancers, and in 12% of PTEN normal but in 26% of PTEN deleted cancers.

Conclusions: This study identifies ROCK1 expression associated with prognosis in prostate cancer.

Methods: We tested ROCK1 expression in 12 427 prostate cancer specimens and followed PSA recurrence after prostatectomy.

## INTRODUCTION

Prostate cancer is the most prevalent cancer in men in Western societies [1]. Although the majority of prostate cancers behave in an indolent manner, a small subset is highly aggressive and requires extensive treatment [2]. Established preoperative prognostic parameters are limited to Gleason grade and tumor extent on biopsies, serum prostate-specific antigen (PSA), levels and clinical stage. These parameters are statistically strong, but not sufficient to enable optimal treatment decisions in every patient. It is, thus, hoped that a better understanding of disease biology will eventually lead to the identification of clinically applicable molecular markers that enable a more reliable prediction of prostate cancer aggressiveness.

Rho-associated coiled-coil containing kinase 1 (ROCK1) belongs to the family of so-called AGC kinases comprising more than 60 evolutionary related serin/threonine protein kinases including important anti-cancer targets such as AKT, p70S6 kinase, or GSK-3ß [3]. ROCK1 is a critical regulator of the shape and motility of mammalian cells by acting on the cytoskeleton. ROCK1 indirectly controls polymerization and depolymerization of actin filaments through activation of its downstream targets LIM kinase and cofilin and promotes contraction of actin fibers through phosphorylation of myosin light chains [3]. Besides its impact on cell motility, ROCK1 has also been implicated in cell growth and cell-cell adhesion. Earlier work has demonstrated that ROCK1 can stimulate PTEN activity [4], inhibit premature centriole separation during cytokinesis [5], antagonize insulin-like growths factor signaling, and facilitate disruption of E-cadherin dependent cell-cell contacts [6]. Two homologs have been identified, ROCK1 and ROCK2, which are encoded by distinct genes [3] but show a high amino acid sequence similarity especially in the kinase domain [7]. Particularly ROCK1 appears to be relevant in many human cancer types, as immunohistochemistry studies reported associations between adverse tumor features and increased ROCK1 protein levels in breast [8], colorectal [9], and gastric cancers [10] as well as in osteosarcomas [11]. Moreover, ROCK1 is a putative drug target as ROCK1 inhibitors are currently tested in clinical trials on advanced solid cancers (e.g. NCT01585701).

ROCK1 may be also implicated in prostate cancer biology. ROCK1 is androgen responsive [12], inhibits apoptosis [13] and promotes cell motility and proliferation [14] in prostate cancer cells. Genetic variants of RhoA and ROCK1 genes have been suggested as susceptibility factors for prostate cancer development [15]. One recent study on 56 prostate cancers and adjacent normal tissue reported a higher level of ROCK1 protein expression in tumor glands as compared to normal tissues [16]. To better understand the potential clinical impact of ROCK1 protein expression in prostate cancer we took advantage of our large prognosis tissue microarray (TMA) with its attached database on clinical, pathological and molecular data and studied patterns of ROCK1 expression in more than 12 000 prostate cancer patients by immunohistochemistry.

## RESULTS

A total of 10 613/12 427 (85.4%) tumor samples were interpretable in our TMA analysis. Reasons for non-informative cases included lack of tissue samples or absence of unequivocal cancer tissue in the TMA spot in 1814/12 427 (14.6%) tumors.

### ROCK1 expression in normal and cancerous prostate tissues

**Figure 1:**
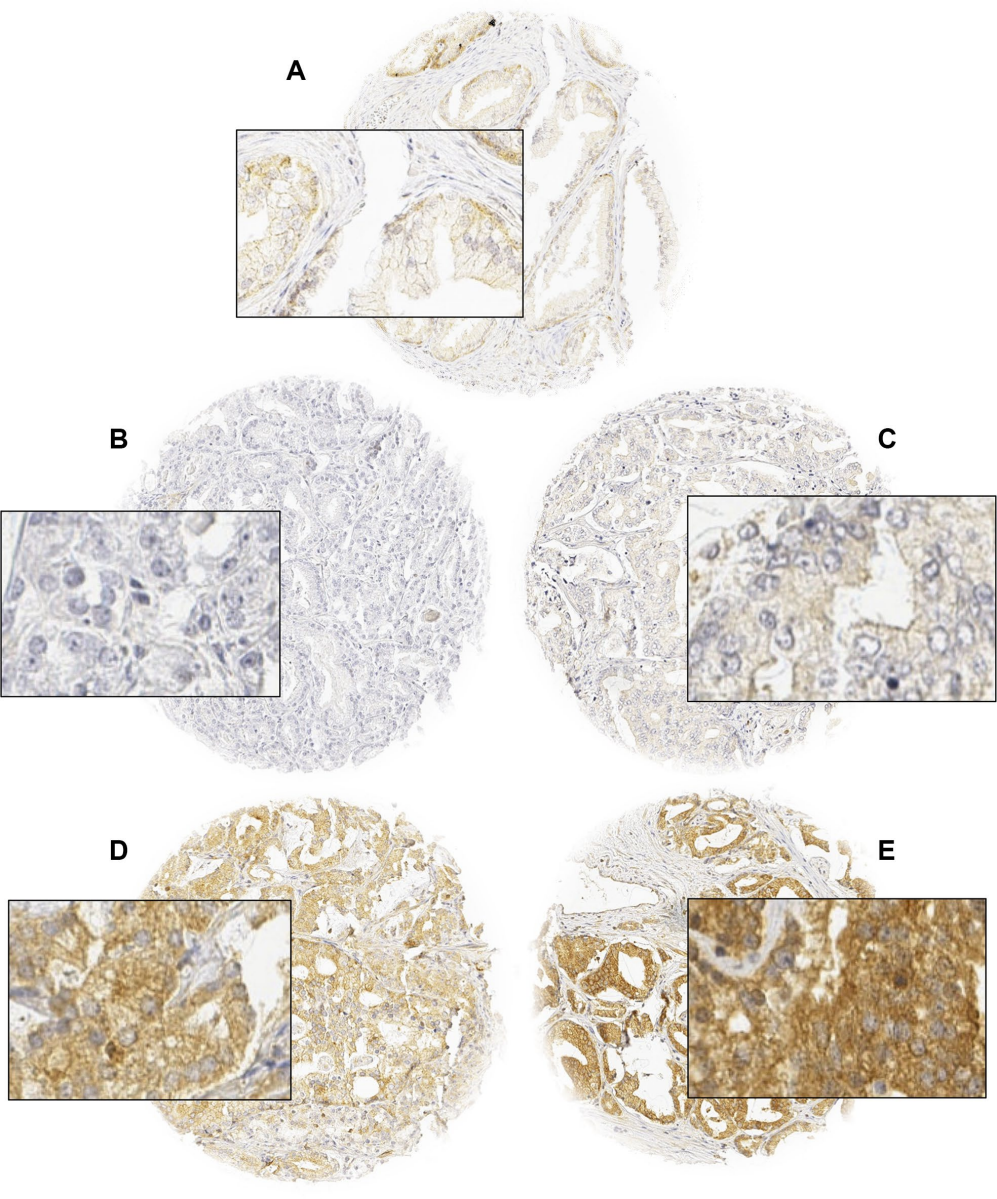
Representative images of normal (**A**) and cancerous glands (**B**–**E**) with negative (**B**), weak (**C**), moderate (**D**), and strong (**E**) ROCK1 staining. Spot size is 600 μm at 100 / 400x of originals.

Normal prostate glands showed weak cytoplasmic staining of luminal and basal cells. In cancers, at least weak ROCK1 staining was found in 92% of the cases, and was considered weak in 22%, moderate in 53%, and strong in 18% of tumors (Table 1). Samples with adjacent normal and cancerous glands revealed that staining was typically stronger in cancer cells as compared to normal prostate glands. Representative images of ROCK1 immunostainings in normal and cancerous glands are shown in Figure 1.

**Table 1:** Association between ROCK1 staining and prostate cancer phenotype in all cancers.

**Parameter**	**ROCK1 (%)**					
	**N**	**Negative**	**Weak**	**Moderate**	**Strong**	**P**
**All cancers**	10 613	8.0	21.5	52.6	17.9	
**Tumor stage**						<0.0001
pT2	6893	8.9	22.6	53.5	15.1	
pT3a	2335	6.9	19.8	52.3	21.0	
pT3b-pT4	1340	5.1	19.0	48.7	27.2	
**Gleason grade**						<0.0001
≤3+3	2336	12.6	29.5	50.2	7.7	
3+4	5830	7.1	20.0	54.8	18.1	
3+4 Tertiary 5	383	6.3	18.8	55.4	19.6	
4+3	1099	5.2	18.4	48.9	27.6	
4+3 Tertiary 5	609	3.9	15.8	52.1	28.2	
≥4+4	521	5.2	17.3	48.9	28.6	
**Quantitative Gleason grade**						<0.0001
≤3+3	2336	12.6	29.5	50.2	7.7	
3+4 ≤5%	1545	8.1	22.5	54.6	14.9	
3+4 6–10%	1514	7.7	19.9	55.7	16.7	
3+4 11–20%	1251	7.1	18.1	54.4	20.4	
3+4 21–30%	681	5.3	17.2	55.4	22.2	
3+4 31–49%	536	5.6	20.7	50.6	23.1	
3+4 Tertiary 5	383	6.3	18.8	55.4	19.6	
4+3 50–60%	462	5.6	20.1	50.2	24.0	
4+3 61–100%	609	3.9	15.8	52.1	28.2	
4+3 Tertiary 5	513	4.7	16.0	47.0	32.4	
≥4+4	521	5.2	17.3	48.9	28.6	
**Lymph node metastasis**						<0.0001
N0	5963	7.1	19.3	53.1	20.5	
N+	607	4.9	16.3	48.8	30.0	
**Preoperative PSA level (ng/ml)**						0.0111
<4	1324	6.1	21.2	53.9	18.8	
4–10	6348	7.8	21.0	53.3	17.9	
10–20	2106	9.4	22.9	50.6	17.0	
>20	720	8.8	22.2	49.7	19.3	
**Surgical margin**						<0.0001
Negative	8463	8.0	21.7	53.3	17.0	
Positive	1959	7.8	21.4	49.4	21.4	

### *TMPRSS2:ERG* fusion status and ERG protein expression

Data on both ERG FISH and IHC were available from 5,976 cancers, and a concordant result (ERG IHC positive and break by FISH or ERG IHC negative and missing break by FISH) was found in more than 95% of the examined cancers. Increased ROCK1 expression was associated with *TMPRSS2:ERG* fusion positive prostate cancers (Supplementary Figure 1). For example, moderate to strong ROCK1 staining was seen in 63% of ERG IHC negative, but in 82% of ERG IHC positive cancers.

### Tumor phenotype and PSA recurrence

**Figure 2:**
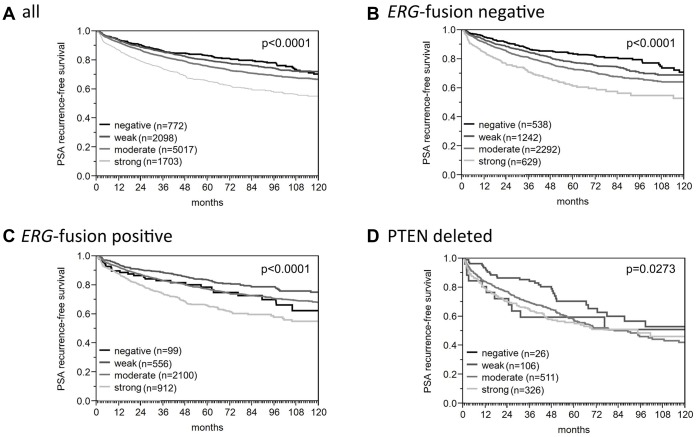
Association between ROCK1 expression and biochemical recurrence in (**A**) all cancers, (**B**) *ERG*-fusion negative cancers, (**C**) *ERG*-fusion positive cancers, (**D**) PTEN deleted cancers.

Increased ROCK1 expression was significantly associated with advanced tumor stage, high classical and quantitative Gleason grade, positive nodal stage, positive surgical margin (p<0.0001 each), and high preoperative PSA level (p=0.0111; Table 1). For example, strong ROCK1 expression was found in 27.2% of pT3b-pT4 tumors and 30% of nodal-positive cancers, but only in 15% of pT2 cancers and 20% of nodal-negative cancers (p<0.0001 each). Most of these associations held also true in subset analyses of ERG negative or ERG positive cancers (Supplementary Table 1 and 2). Follow-up data were available for 9,590 patients with interpretable ROCK1 immunostaining. ROCK1 expression was associated with early PSA recurrence and (Figure 2A). This also hold true for the subsets of *ERG*-fusion negative and positive cancers (Figures 2B–2C) as well as in the subset of PTEN deleted (Figure 2D) and PTEN wild type cancers (p<0.0001, data not shown). To compare the prognostic impact of ROCK1 expression and the Gleason grade, further subset analyses were performed in cancers with identical classical and quantitative Gleason grade (Supplementary Figure 2). It showed that ROCK1 staining lacked significant prognostic impact in any group defined by classical or quantitative Gleason grade.

### Association to patient age

ROCK1 upregulation was weakly linked to higher patient age when all tumors were jointly analyzed. The fraction of cancers with strong ROCK1 positivity increased from 13.4% in patients aged below 50 years to 20.5% in elderly patients (>70 years, p<0.0001). A subset analysis of revealed that this association was solely driven by cancers harboring TMPRSS2:ERG fusions (12.7% – 34.0%, p<0.0001) but was absent in fusion negative tumors (12.5% – 12.4%, p=0.4249). All data are summarized in Supplementary Table 3.

### Multivariate analysis

Four different multivariate analyses were performed to evaluate the clinical relevance of ROCK1 expression in different scenarios (Supplementary Table 4). Scenario 1 evaluated all postoperatively available parameters including pT, pN, surgical margin status, preoperative PSA value and Gleason grade obtained on the prostatectomy specimen. In scenario 2, all postoperatively available parameters except pN were included. The rationale for this approach was that the indication and extent of lymph node dissection is not standardized in the surgical therapy of prostate cancer and may introduce a bias towards high-grade cancers. Two additional scenarios were to model the preoperative situation as much as possible. Scenario 3 included ROCK1 expression, preoperative PSA, clinical tumor stage (cT stage) and Gleason grade obtained on the prostatectomy specimen. Since postoperative determination of a tumor’s Gleason grade is “better” than the preoperatively determined Gleason grade (subjected to sampling errors and consequently under grading in more than one third of cases [17]) this parameter was replaced by the preoperative Gleason grade obtained on the original biopsy in Scenario 4. ROCK1 expression provided significant prognostic value beyond the established parameters in all of the described scenarios, particularly in the preclinical scenario 4. This also held true for the subgroups of ERG negative and ERG positive cancers. The cox proportional hazard ratio of PSA recurrence-free survival for patients with strong versus negative ROCK1 expression was in univariate analysis a moderate 2.04 (Supplementary Table 5).

### Androgen receptor

**Figure 3:**
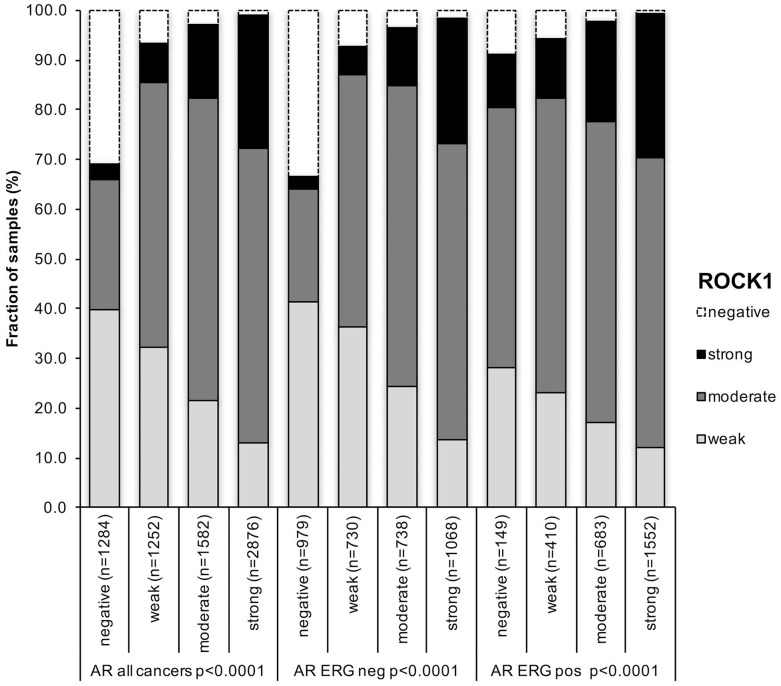
Association between positive ROCK1 staining and androgen-receptor (AR) status in all cancer, ERG fusion negative and ERG fusion positive cancers.

To estimate the impact of AR on ROCK1 expression, we used AR expression data from a previous study [18], Data on both ROCK1 and AR were available from 6994 cancers. There was a strong positive association between AR expression and ROCK1 expression in all cancers as well as in subsets of ERG negative and ERG positive cancers (Figure 3).

### Association with cell proliferation

ROCK1 was significantly associated with increased cell proliferation as measured by Ki67-LI in all cancers (p<0.0001). The average Ki67-LI increased from 1.62±0.12 in cancers lacking ROCK1 expression to 2.28±0.07 (weak), 2.93±0.05 (moderate) and to 3.72±0.08 in cancers with strong ROCK1 expression (Table 2). This association held also true in tumor subsets with identical Gleason score.

**Table 2:** Association between ROCK1 expression and Ki67-labeling index in all cancers.

**Subset**	**ROCK1**	**N**	Ki67-LI*	**P**
All	Negative	506	1.3±0.12	p<0.0001
	Weak	1391	2.3±0.07	
	Moderate	3279	2.9±0.05	
	Strong	1044	3.7±0.08	
Gleason ≤3+4	Negative	448	1.2±0.11	p<0.0001
	Weak	1167	2.1±0.07	
	Moderate	2666	2.8±0.04	
	Strong	737	3.3±0.08	
Gleason ≥4+3	Negative	55	1.9±0.50	p<0.0001
	Weak	215	3.0±0.26	
	Moderate	598	3.7±0.15	
	Strong	299	4.7±0.22	
PTEN normal	Negative	165	1.8±0.21	p<0.0001
	Weak	726	2.5±0.10	
	Moderate	1808	3.1±0.06	
	Strong	540	3.8±0.12	
PTEN deletion	Negative	17	3.1±0.70	p=0.1086
	Weak	78	3.1±0.33	
	Moderate	376	3.6±0.15	
	Strong	214	3.9±0.20	

* Mean ± standard error of the mean

### Chromosomal deletions

**Figure 4:**
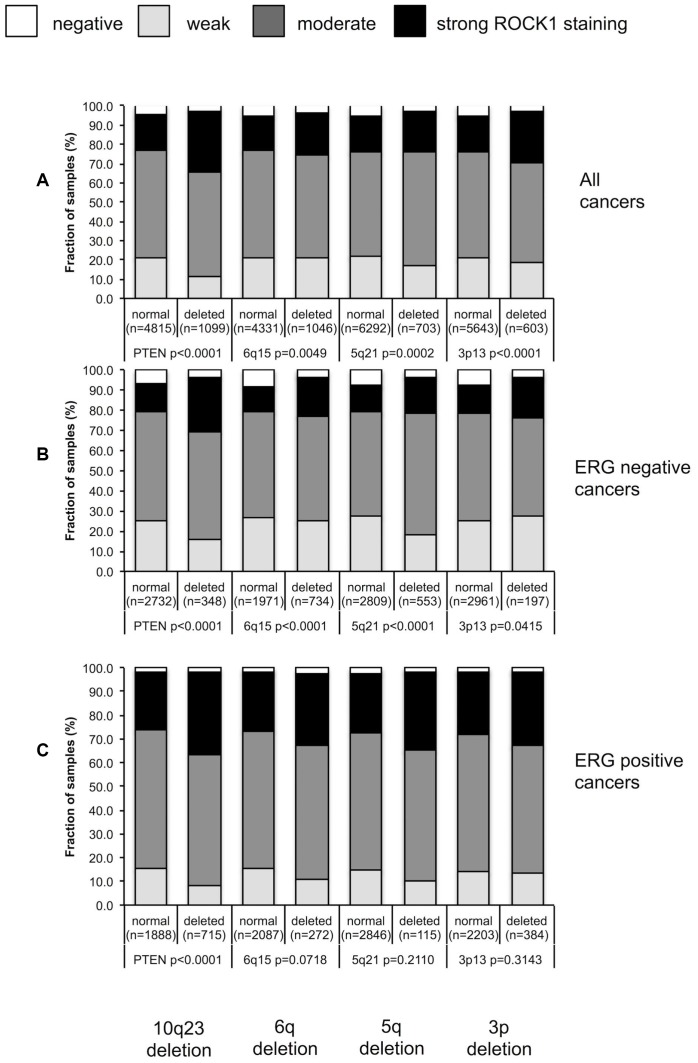
Association between ROCK1 staining and 10q23 *(PTEN)*, 5q21 *(CHD1)*, 6q15 *(MAP3K7)*, 3p13 (*FOXP1*) deletions in (**A**) all cancers, (**B**) ERG negative cancers and (**C**) in ERG positive cancers.

For PTEN, 6q15, 5q21 and 3p13, there was a tendency towards a higher level of ROCK1 immunostaining if deletions were present (Figure 4). This became particularly clear in ERG negative cancers (p<0.05 for all deletions; Figure 4B). In ERG positive cancers, this association reached statistical significance only for PTEN (p<0.0001; Figure 4C).

## DISCUSSION

The results of this study demonstrate that overexpression of ROCK1 is associated with adverse tumor features and early biochemical recurrence in prostate cancer.

That ROCK1 staining was generally higher in cancers than in tumor adjacent normal prostatic tissue argues for a role of ROCK1 up regulation in prostate cancer development. Positive ROCK1 staining was seen in more than 90% of our 10,613 interpretable cancers, including 22% cancers with weak, 53% cancers with moderate and 18% with strong staining. Higher ROCK1 levels in cancer, than in normal epithelium was also described in an immunohistochemistry study by Kroiss et al. [16] reporting 100% positive staining in 56 cancers, including 14% cancers with low, 57% with moderate, and 29% with strong staining. The slightly higher fraction of cancers with strong staining in the study by Kroiss et al. is mostly likely due to the use of a different antibodies (Genetex GTX113266, rabbit polyclonal) and staining protocols.

The association with unfavorable tumor phenotype and poor clinical outcome in our patients argues for a role of ROCK1 overexpression in prostate cancer progression. While comparable immunohistochemistry data on the prognostic role of ROCK1 expression in prostate cancer are lacking, analysis of mRNA expression data from 85 prostate cancers from Taylor et al. [19] (raw data via GEO GSE21032) confirmed ROCK1 up-regulation in 21 recurrent (average mRNA expression level: 8.3) as compared to disease-free cancers (average 8.1, p=0.0263). A clinically relevant role of ROCK1 overexpression in prostate cancer is also consistent with reports from several other cancer types, where high ROCK1 expression was associated with tumor aggressiveness [8–10]. Schmidt et al. reported immunohistochemically detectable expression of ROCK’s upstream regulator RhoA to occur in >90% of prostate cancers and described a link between high level RhoA expression to large tumor size and poor prognosis [20]. Increased cancer aggressiveness in case of ROCK1 and/or RhoA overexpression fits very well with the known role of the Rho/Rock signaling pathway as a regulator of the actin cytoskeleton dynamics, and therefore, of various critical tumor-relevant processes such as cell motility, growth, dell division and cell survival. In vitro- and in vivo-studies with ROCK1 inhibitors suggest that ROCK1 activation increases motility, invasiveness and metastasis through accelerated actin filament remodeling and/or through indirect effects on the stiffness of the extracellular matrix, for example in mouse models of breast cancer bone metastasis [21], in ovarian cancer cells [22], and in lung cancer cells [23]. A role for tumor cell proliferation and angiogenesis is also assumed based on the anti-proliferative and anti-angiogenic effects of ROCK1 inhibition in lung cancer cells [24]. Moreover, ROCK1 has been shown to activate proliferation-promoting oncogenes such as c-myc by direct phosphorylation in breast cancer [21] and in prostate cancer [14]. An anti-apoptotic effect of ROCK1 has been reported in bladder cancer [25] and leukemia [26], and it was demonstrated that ROCK1 can disrupt the apoptotic-signaling cascade through inhibitory binding to Erk1/2 kinase [26].

The molecular database attached to our TMA allowed us to compare ROCK1 expression with other important molecular alterations occurring in prostate cancer. More than half of all prostate cancers, particularly those of young patients, harbor fusions connecting the androgen-regulated *TMPRSS2* gene with the transcription factor *ERG* [18, 27]*.* These fusions result in an androgen-dependent overexpression of ERG [28] eventually leading to an altered expression of more than 1,600 genes in prostate epithelial cells [29]. The significant up-regulation of ROCK1 in cancers harboring the *TMPRSS2:ER*G fusion fits with an earlier report describing increased expression of ROCK’s upstream regulator Rho guanine diphosphate dissociation inhibitor beta (ARHGDIB) in ERG positive prostate cancers [30]. Of note, ARHGDIB up-regulation has also been reported from breast cancers [31] where it promotes invasiveness [32]. Interestingly, we found that ROCK1 up-regulation was linked to higher patient age exclusively in cancers harboring the ERG fusion, suggesting that the consequences of ERG fusion may vary with age. The positive association between AR and ROCK1 expression further supports recent work suggesting a regulatory loop involving both proteins. Two studies demonstrate that Rho/ROCK signaling activity is increased in response to androgen stimulation [20], while the androgen-regulated micro-RNA-135a controls ROCK1 expression [16].

Genomic deletions at various chromosomal loci represent the second most frequent type of genetic alterations next to *TMPRSS2:ERG* fusions. The significant association between high ROCK1 expression and several important deletions fits with earlier studies showing that perturbations in the actin filament homeostasis, which can occur as a consequence of ROCK1 deregulation [33], promote the development of general genetic instability including double strand breakage and deletions. In human pluripotent stem cells, which often acquire chromosomal aberrations in culture, replicative stress, defective chromosome condensation and aneuploidy had been associated with altered levels of actin cytoskeletal genes [34]. Those associations between ROCK1 and most deletions were less common in ERG positive than in ERG negative cancers may be due to the different microenvironment in ERG positive cells. Alternatively, this may be due to experimental issues caused by the general ROCK1 up-regulation in ERG positive cancers. In cancers with a higher average expression, the distinction of subtle expression differences may become more difficult in bright field immunohistochemistry. The particularly strong association between ROCK1 expression and *PTEN* deletions is likely to be due to the known PTEN/ROCK1 interaction. ROCK1 has been shown to modify PTEN activity in several studies, although with conflicting results describing either activation [4] or down-regulation of PTEN [35]. The data of this study suggest that ROCK1 expression may represent a potentially clinically useful prognostic marker in prostate cancer. Of note, the independent prognostic role of ROCK1 expression was even retained if the strongest established prognostic parameters were included, such as pT and pN stage, which are not available at the moment when therapeutic decisions are taken. Moreover, ROCK1 expression had a prognostic impact in all analyzed molecularly defined subgroups. This included cancers harboring deletions of the *PTEN* tumor suppressor, which belongs to the strongest molecular prognostic markers identified in prostate cancer as to yet [36]. That ROCK1 expression lacked prognostic impact in cancers defined by a comparable classical or quantitative Gleason grade demonstrates how difficult it is for a molecular marker to compete with classical histomorphological features. The potential for ROCK1 expression analysis is not compromised by the fact that ROCK1 expression analysis was not better than Gleason grading. Although Gleason grading is a very powerful statistical parameter, it suffers from notorious interobserver heterogeneity, which is in the range of 40% [37]. Accordingly, there is not only a need for better predictors of prostate cancer aggressiveness but also for more reproducible ones. Molecular analysis including one or more molecular parameters may, thus, help to improve standardization of prognosis assessment in the future.

Several drugs targeting ROCK1 are available. Y27632 [38] and Fasudil [39] are selective ROCK inhibitors, which target the ATP-dependent kinase domain of both isoforms ROCK1 and ROCK2. Fasudil is in clinical use in China and Japan in the treatment of cerebral vasospasm [40], pulmonary hypertension [41] and neurodegenerative memory loss [42]. Several studies suggest that it might be also useful for treating cancers [24, 43, 44]. Most recently, two studies found that Fasudil inhibits migration of breast, fibrosarcoma and laryngeal cancer cells [45, 46]. AT13148, a multi-AGC kinase inhibitor targeting ROCK and also various other serin/threonine protein kinases demonstrated potent cytotoxic and anti-proliferative activities in cell lines of human melanomas, gliomas, and various other cancers [47, 48]. AT13148 showed significant antitumor actions also in mouse models of human xenograft pancreatic cancers, where the compound reduced subcutaneous tumor growth and blocked invasion of healthy pancreatic tissue [49]. These promising results lead to the recruitment of patients with advanced solid cancers who are refractory to conventional therapy for a phase I clinical study (NCT01585701). The data from our study suggest a high importance of ROCK1 for prostate cancer biology. This may encourage future work on the effect of ROCK inhibitors in prostate cancer.

## CONCLUSIONS

In summary, the results of our study demonstrate that up-regulation of ROCK1 is common in prostate cancer and is associated with tumor aggressiveness and poor prognosis. Anti-cancer drugs specifically targeting ROCK1 may thus be particularly efficient in prostate cancer. Moreover, ROCK1 measurement, either alone or in combination might be of clinical utility in prostate cancer.

## MATERIALS AND METHODS

### Patients

Radical prostatectomy specimens were from patients, who had been operated between 1992 and 2012 at the Department of Urology and the Martini Clinics at the University Medical Center Hamburg-Eppendorf (Supplementary Table 6). Follow-up was available for a total of 11 613 patients (median 49 months; range: 1 to 276 months). PSA levels were measured following surgery and PSA recurrence was defined as the time point when postoperative PSA was at least 0.2 ng/ml and increasing at subsequent measurements. In addition to the classical Gleason categories, “quantitative” Gleason grading was performed as described before [50]. In brief, for every prostatectomy specimen, the percentages of Gleason 4 patterns in cancerous tissues were estimated during the regular process of pathologic interpretation. The TMA was produced with one 0.6mm core taken from a cancer containing tissue block from each patient. Each TMA block also contained various control tissues, including normal prostate tissue. The TMA is annotated with results on ERG expression, ERG break apart FISH analysis [51] and deletion status of 5q21 (CHD1) [52], 6q15 (MAP3K7) [53], *PTEN* (10q23) [54], 3p13 (FOXP1) [55], Ki67 labeling index (Ki67-LI) data), and androgen receptor (AR) expression [56]. The usage of archived diagnostic leftover tissues for manufacturing of tissue microarrays and their analysis for research purposes as well as patient data analysis has been approved by local laws (HmbKHG, §12a) and by the local ethics committee (Ethics commission Hamburg, WF-049/09). All work has been carried out in compliance with the Helsinki Declaration.

### Immunohistochemistry

Freshly cut TMA sections were stained in one day and in one experiment. Slides were deparaffinized and exposed to heat-induced antigen retrieval for 5 minutes at 121°C in pH 7,8 Tris-EDTA-citrate buffer. Anti- ROCK1 immunohistochemical staining was performed with the rabbit monoclonal antibody clone EP786Y (Abcam ab45171, Cambridge, UK, 1:4050) for 60 min at 37°C. To confirm specificity of clone EP786Y, we partly repeated the staining using a more recent ROCK1 antidody that had been validated in ROCK1 knock-out cell lines (Abcam ab134181, clone EPR683Y). The identical staining pattern that was observed with both antibodies is compatible with specificity for the ROCK1 protein (Supplementary figure 3). ROCK1 staining was typically cytoplasmic and slightly nuclear. As no significant heterogeneity in ROCK1 staining was seen in TMA spots, the precentage of positive cells was not considered. Instead, the overall staining intensity (0, 1+, 2+, and 3+) of cancer cells was recorded for each tissue spot. Only one trained person analyzed all tissue spots manually. We do not regard this as a limitation of our study. We have earlier demonstrated that manual and automated image analysis of large scale TMAs yield comparable results [57] and that possible misdiagnosis of individual tissue spots (e.g. false classification of tumor and normal tissue) does not significantly impact the overall study outcome [58].

### Statistics

Contingency tables and the chi²-test were performed to search for associations between molecular parameters and tumor phenotype. Kaplan-Meier curves were tested by the log-rank test to detect significant differences between groups. Cox proportional hazards regression analysis was applied to test the statistical independence and significance between pathological, molecular and clinical variables. All calculations were done with JMP 12 (SAS Institute Inc., NC, USA).

## Supplementary Material

Supplementary Figures

Supplementary Tables
