# Supplementary material for: Up regulation of Rho-associated coiled-coil containing kinase1 (ROCK1) is associated with genetic instability and poor prognosis in prostate cancer
Source: Aging (Albany NY). 2019 Sep 25;11(18):7859–79. doi: 10.18632/aging.102294 (PMC6781985; doi:10.18632/aging.102294)
Supplement: Supplementary Figures [file aging-11-102294-s002.pdf]

SUPPLEMENTARY FIGURES

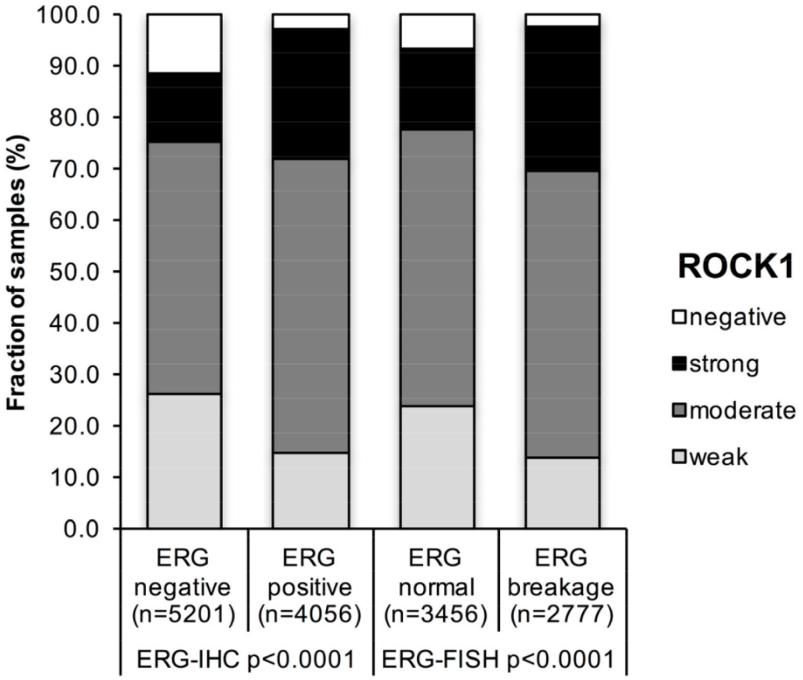

Supplementary Figure 1. Association between positive ROCK1 staining and ERG-status (IHC/FISH).

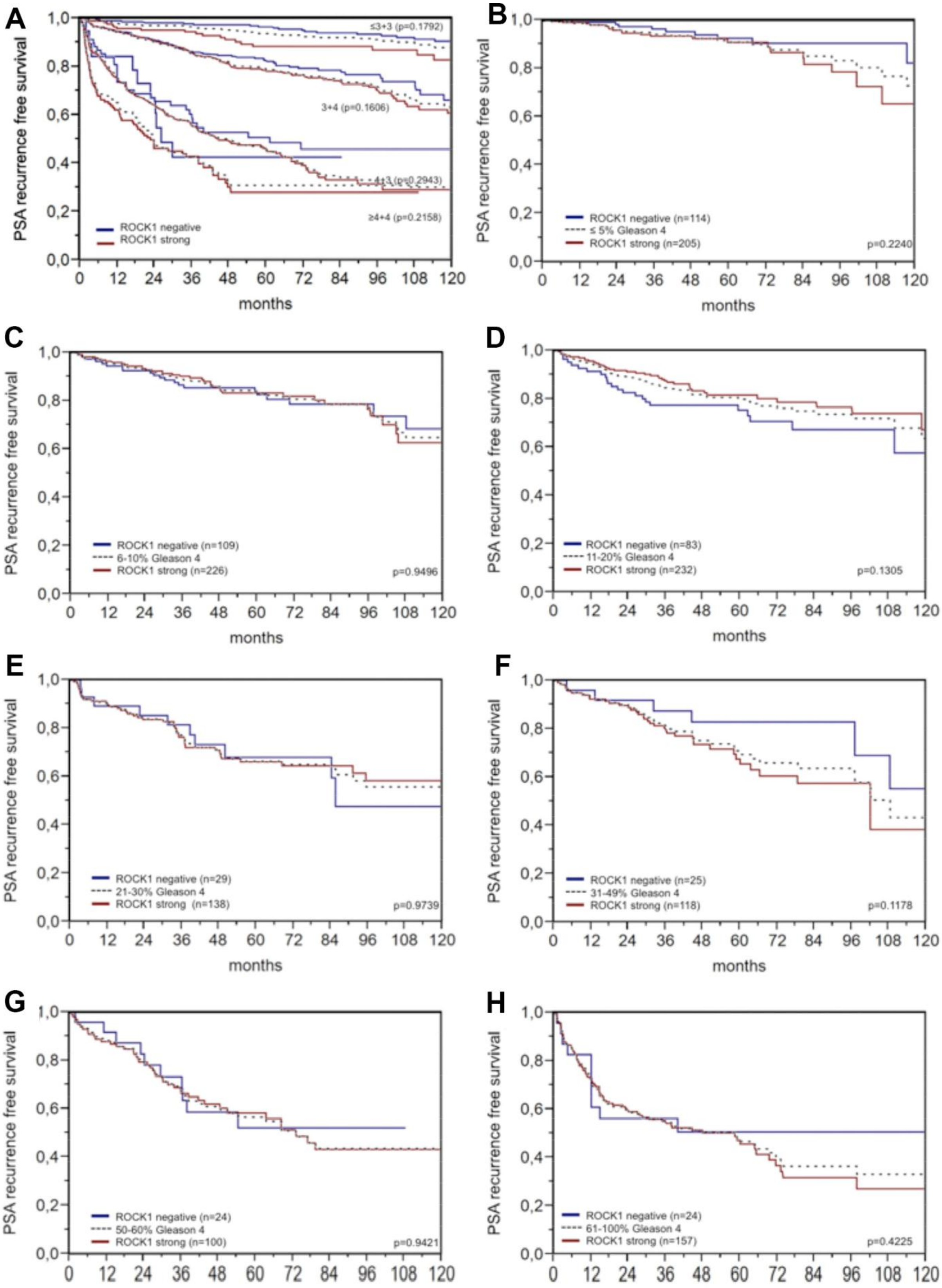

Supplementary Figure 2. Association between ROCK1 expression (negative versus strong) and biochemical recurrence in (**A**) classic Gleason grade, (**B**) <5% Gleason 4, (**C**) 6–10% Gleason 4, (**D**) 11-20% Gleason 4, (**E**) 21-30% Gleason 4, (**F**) 31-49% Gleason 4, (**G**) 50–60% Gleason 4, and (**H**) ≥ 61% Gleason 4 pattern.

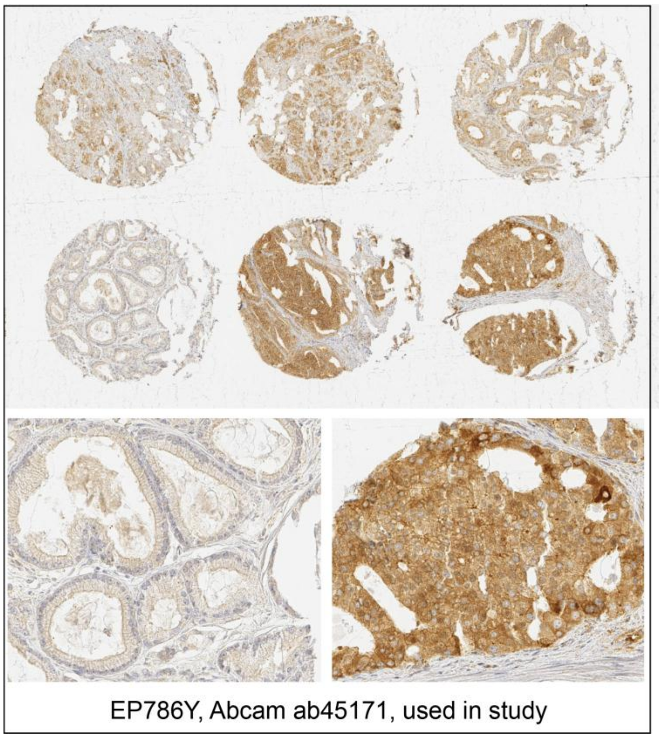

Supplementary Figure 3A. Examples of immunostainings using anti-Rock1 antibody EP786Y that was used in the current study.

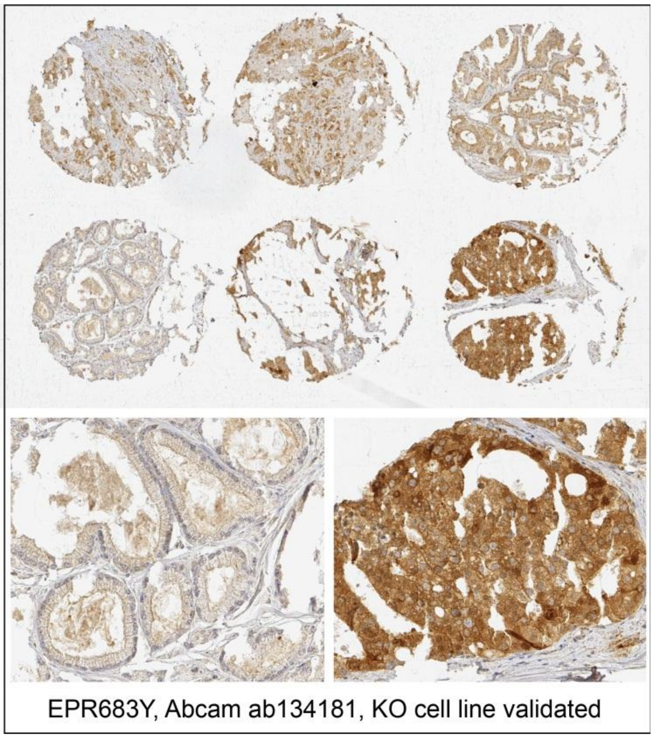

Supplementary Figure 3B. Examples of immunostainings using anti-Rock1 antibody EPR683Y. Note the identical staining pattern as compared to EP786Y (Supplementary Figure 3A). Specificity of EPR683Y was validated by analysis of wildtype and ROCK1 knock out cells.
