# Supplementary material for: Up regulation of Rho-associated coiled-coil containing kinase1 (ROCK1) is associated with genetic instability and poor prognosis in prostate cancer
Source: Aging (Albany NY). 2019 Sep 25;11(18):7859–79. doi: 10.18632/aging.102294 (PMC6781985; doi:10.18632/aging.102294)
Supplement: Supplementary Tables [file aging-11-102294-s001.pdf]

SUPPLEMENTARY TABLES

Supplementary Table 1. Association between ROCK1 staining and prostate cancer phenotype in ERG *negative* cancers.

Parameter	N	ROCK1 (%)				P
		Negative	Weak	Moderate	Strong	
	5201	11.3	26.1	49.3	13.3	
**Tumor stage**						<0.0001
pT2	3508	12.4	26.4	49.5	11.7	
pT3a	1031	10.3	26.6	49.5	13.7	
pT3b-pT4	644	7.0	23.6	48.1	21.3	
**Gleason grade**						<0.0001
≤3+3	1042	18.3	34.5	41.1	6.0	
3+4	2798	10.6	25.3	51.4	12.6	
3+4 Tert.5	227	8.4	22.0	54.6	15.0	
4+3	574	7.5	22.6	50.0	19.9	
4+3 Tert.5	330	5.5	20.0	51.5	23.0	
≥4+4	302	5.3	21.9	50.3	22.5	
**Quantitative Gleason grade**						
≤3+3	1042	18.3	34.5	41.1	6.0	<0.0001
3+4 ≤5%	766	11.1	26.6	51.8	10.4	
3+4 6–10%	721	11.4	25.2	50.9	12.5	
3+4 11–20%	624	11.5	23.7	51.8	13.0	
3+4 21–30%	315	9.2	23.5	53.0	14.3	
3+4 31–49%	273	8.8	27.8	46.5	16.8	
3+4 Tert.5	227	8.4	22.0	54.6	15.0	
4+3 50–60%	236	9.3	22.9	51.3	16.5	
4+3 61–100%	330	5.5	20.0	51.5	23.0	
4+3 Tert.5	281	6.8	21.0	48.4	23.8	
≥4+4	302	5.3	21.9	50.3	22.5	
**Lymph node metastasis**						<0.0001
N0	3003	10.5	23.6	51.1	14.8	
N+	288	6.6	20.1	46.2	27.1	
**Preoperative PSA level (ng/ml)**						0.3238
<4	552	9.4	26.1	49.8	14.7	
4-10	3068	10.9	26.0	49.9	13.2	
10-20	1134	13.1	26.2	48.7	12.0	
>20	402	11.7	25.6	47.0	15.7	
**Surgical margin**						0.0071
Negative	4154	11.3	26.1	50.1	12.5	
Positive	960	10.9	26.7	45.8	16.6	

Supplementary Table 2. Association between ROCK1 staining and prostate cancer phenotype in ERG *positive* cancers.

Parameter	N	ROCK1 (%)				P
		Negative	Weak	Moderate	Strong	
	4056	2.7	14.9	57.1	25.3	
**Tumor stage**						<0.0001
pT2	2421	2.7	16.0	60.1	21.2	
pT3a	1068	2.4	13.1	55.5	28.9	
pT3b-pT4	550	2.9	13.6	46.5	36.9	
**Gleason grade**						<0.0001
≤3+3	827	3.7	23.8	61.7	10.8	
3+4	2396	2.4	13.2	59.0	25.3	
3+4 Tert. 5	124	0.8	14.5	54.0	30.6	
4+3	409	1.7	11.5	47.4	39.4	
4+3 Tert. 5	225	1.8	9.3	48.9	40.0	
≥4+4	163	3.1	9.8	47.2	39.9	
**Quantitative Gleason grade**						
≤3+3	827	3.7	23.8	61.7	10.8	<0.0001
3+4 ≤5%	591	2.9	17.9	58.9	20.3	
3+4 6-10%	642	2.5	11.2	63.2	23.1	
3+4 11-20%	513	1.9	11.3	56.9	29.8	
3+4 21-30%	300	1.7	10.3	57.0	31.0	
3+4 31-49%	215	2.3	13.0	53.0	31.6	
3+4 Tert.5	124	0.8	14.5	54.0	30.6	
4+3 50-60%	181	0.6	16.6	49.2	33.7	
4+3 61-100%	225	1.8	9.3	48.9	40.0	
4+3 Tert.5	186	2.2	7.0	44.6	46.2	
≥4+4	163	3.1	9.8	47.2	39.9	
**Lymph node metastasis**						
N0	2315	1.9	13.3	55.5	29.3	0.1389
N+	256	3.1	10.9	51.2	34.8	
**Preoperative PSA level (ng/ml)**						0.4117
<4	562	1.6	14.9	58.4	25.1	
4–10	2470	2.8	14.3	57.9	25.0	
10–20	729	3.0	16.3	54.5	26.2	
>20	248	2.4	17.3	52.0	28.2	
**Surgical margin**						0.0091
Negative	3190	2.5	15.2	58.2	24.1	
Positive	791	3.0	14.3	53.0	29.7	

Supplementary Table 3. Association between ROCK1 staining and patient age in all cancers, ERG *negative* cancers and in ERG *positive* cancers.

	age	n	ROCK1 IHC result (%)				p
			negative	weak	moderate	strong	
all cancers	<50	292	6.2	18.8	61.6	13.4	<0.0001
	50–59	2670	6.7	20.1	55.4	17.8	
	60–70	6229	8.3	22.6	51.3	17.7	
	>70	1548	7.6	19.6	52.3	20.5	
ERG negative	<50	80	10.0	23.8	53.8	12.5	0.4249
	50–59	1007	10.6	25.4	50.6	13.3	
	60–70	2638	12.5	28.3	47.1	12.1	
	>70	722	11.1	27.4	49.4	12.0	
ERG positive	<50	134	3.0	13.4	70.9	12.7	<0.0001
	50–59	1045	2.4	16.6	60.0	21.1	
	60–70	2166	3.3	17.5	54.2	25.0	
	>70	444	2.3	9.9	53.8	34.0	

Supplementary Table 4. Multivariate analysis including ROCK1 expression in all cancers, ERG negative and ERG positive cancers.

Subset	Scenario	N	P of Cox hazard ratio for PSA recurrence-free survival							
			Preoperative PSA-Level	pT-stage	cT-stage	Gleason grade prostatectomy	Gleason grade biopsy	pN-stage	R- stage	ROCK1-expression
**All**	1	5789	<0.0001	<0.0001	-	<0.0001	-	<0.0001	0.0075	0.0029
	2	9373	<0.0001	<0.0001	-	<0.0001	-	-	<0.0001	0.0004
	3	9224	<0.0001	-	<0.0001	<0.0001	-	-	-	0.0008
	4	9103	<0.0001	-	<0.0001	-	<0.0001	-	-	<0.0001
**ERG- *negative***	1	2912	<0.0001	<0.0001	-	<0.0001	-	0.0013	0.4537	0.0165
	2	4600	<0.0001	<0.0001	-	<0.0001	-	-	0.0068	0.0134
	3	4550	<0.0001	-	<0.0001	<0.0001	-	-	-	0.0024
	4	4495	<0.0001	-	<0.0001	-	<0.0001	-	-	<0.0001
**ERG- *positive***	1	2264	0.0009	<0.0001	-	<0.0001	-	0.0236	0.0335	0.017
	2	3582	0.0009	<0.0001	-	<0.0001	-	-	0.0002	0.0018
	3	3504	<0.0001	-	<0.0001	<0.0001	-	-	-	0.0067
	4	3455	<0.0001	-	<0.0001	-	<0.0001	-	-	<0.0001

Scenario 1 includes all postoperatively available parameters (pathological tumor (pT) stage, lymph node (pN), surgical margin (R) status, preoperative PSA value and Gleason grade obtained after the morphological evaluation of the entire resected prostate. Scenario 2 excluded the nodal status from analysis. Scenario 3 included preoperative PSA, clinical tumor (cT) stage and Gleason grade obtained on the prostatectomy specimen. In scenario 4, the preoperative Gleason grade obtained on the original biopsy was combined with preoperative PSA, and cT stage.

Supplementary Table 5. Cox proportional hazards for PSA recurrence-free survival after prostatectomy of established preoperative prognostic parameter and ROCK1 expression.

Variable	Univariate analysis	Multivariate analysis
**Gleason grade biopsy**		
3+4 vs. ≤3+3	2.20 (2.00-2.41) ***	1.81 (1.63-2.01) ***
4+3 vs. 3+4	1.85 (1.66-2.05) ***	1.65 (1.47-1.86) ***
≥4+4 vs. 4+3	1.48 (1.32-1.66) ***	1.33 (1.17-1.52) ***
**cT-stage**		
≥T2c vs. T1c	3.95 (3.24-4.76) ***	2.11 (1.70-2.59) ***
**Preoperative PSA-level (ng/µl)**		
4-10 vs. <4	1.26 (1.12-1.42) **	1.34 (1.15-1.57) **
10-20 vs. 4-10	2.08 (1.93-2.25) ***	1.58 (1.43-1.74) ***
>20 vs. 10-20	1.95 (1.76-2.16)***	1.65 (1.44-1.88) ***
**ROCK1 expression**		
Strong vs. negative	2.04 (1.72-2.44) ***	1.55 (1.30-1.86) ***
ERG negative subset	2.41 (1.91-3.07) ***	-
ERG positive subset	1.61 (1.11-2.42) *	-

Confidence interval (95%) in brackets; asterisk indicate significance level: * p≤0.05, ** p≤0.001, *** p≤0.0001.

Supplementary Table 6. Pathological and clinical data of the arrayed prostate cancers.

	Study cohort on TMA*	Biochemical relapse among categories
**Follow-up**		
n	11 665	2769 (23.7%)
Mean / Median (month)	62 / 49	-
**Age (y)**		
≤50	334	81 (24.3%)
51-59	3061	705 (23%)
60-69	7188	1610 (22.4%)
≥70	1761	370 (21%)
**Pretreatment PSA (ng/ml)**		
<4	1585	242 (15.3%)
4-10	7480	1355 (18.1%)
10-20	2412	737 (30.6%)
>20	812	397 (48.9%)
**pT stage (AJCC 2002)**		
pT2	8187	1095 (13.4%)
pT3a	2660	817 (30.7%)
pT3b	1465	796 (54.3%)
pT4	63	51 (81%)
**Gleason grade**		
≤3+3	2848	234 (8.2%)
3+4	6679	1240 (18.6%)
3+4 Tert.5	433	115 (26.6%)
4+3	1210	576 (47.6%)
4+3 Tert.5	646	317 (49.1%)
≥4+4	596	348 (58.4%)
**pN stage**		
pN0	6970	1636 (23.5%)
pN+	693	393 (56.7%)
**Surgical margin**		
Negative	9990	1848 (18.5%)
Positive	2211	853 (38.6%)

* Numbers do not add up to 12 427 in all categories due to missing values.
AJCC American Joint Committee on Cancer
